# MASLD: time to optimise regional research priorities

**DOI:** 10.1016/j.lansea.2026.100732

**Published:** 2026-02-19

**Authors:** The Lancet Regional Health – Southeast Asia

Metabolic dysfunction-associated steatotic liver disease (MASLD) affects 38% of the population globally, with the southeast Asian region bearing a burden of 33%. India is estimated to have experienced one of the most significant increases in MASLD prevalence between 2010 and 2021. MASLD has the potential to progress to metabolic dysfunction-associated steatohepatitis (MASH) with fibrosis and ultimately cirrhosis. Despite its low risk and relatively slow progression, the asymptomatic nature of the disease often delays diagnosis, leading to adverse consequences such as liver transplantation or even death.

MASLD is often considered a hepatic manifestation of metabolic syndrome, with its epidemiology being driven by the rising burden of diabetes and obesity. Beyond this classical association, south Asian populations face unique difficulties related to the “thin-fat” phenotype, where a distinct category of lean MASLD is prevalent in individuals without obesity but with excess visceral adiposity, often fuelled by diabetes and insulin resistance. Genetic predisposition further exacerbates this risk, with variants such as *PNPLA3* and *TM6SF2* acting as key determinants of lean MASLD in Asian populations. These complexities, along with the absence of appropriate screening algorithms, standardised diagnostic protocols, and validated tools, hinder the diagnosis and management of MASLD in the region.

Current global guidelines recommend image-based detection of steatosis supported by comprehensive anthropometric and biochemical evaluation, which are resource-intensive. Unaddressed, there is a high risk of the condition progressing to fibrosis and cirrhosis. Currently, score-based criteria such as the Fibrosis-4 index (FIB-4), NAFLD Fibrosis Score (NFS), and Steatosis-Associated Fibrosis Estimator (SAFE) are used to identify individuals at risk for fibrosis. Those with higher scores are further evaluated using non-invasive tests like transient elastography (FibroScan), although liver biopsy remains the gold standard. These approaches have not been systematically validated in southeast Asian populations, where screening guidelines and consensus have only recently begun to prioritise individuals with diabetes in certain countries, such as India.

Studies estimating MASLD burden are essential for gathering insights into regional aetiopathogenesis. In this issue of *The Lancet Regional Health – Southeast Asia*, Meghana Arvind and colleagues estimate MASLD prevalence in India through an observational study among employees and their families leveraging the Phenome India cohort. The authors report an estimated age-adjusted prevalence of 38.9%, with 6.3% progressing to fibrosis. Diabetes and obesity emerged as key risk determinants in this cohort, with lean MASLD having higher proportions of people with diabetes. The authors also found that advanced age contributed towards progression of fibrosis and 16 inflammatory markers were elevated, indicating an immune mediated response. Although this study reports findings from a sample of voluntary employees, such large-scale community-based studies have the potential to offer insights into the unique epidemiology and natural history of the disease considering the diverse socio-economic and cultural contexts.

The limited research priority, coupled with high cost and lack of adequate funding, poses a major hurdle to the design and conduct of such studies. These challenges could be addressed by focusing research on affordable and accessible point-of-care diagnostics, such as point-of-care ultrasound (POCUS), and by exploring integration with existing demographic health surveys. POCUS has the potential to improve accessibility and diagnosis in primary care, but its scope has not been expanded to include metabolic disease screening. While several studies from Africa highlight its wide scope across patient management, studies from Asia are limited, which could partly be explained by stringent legal regulation on the use of ultrasound in countries like India.

The need for more targeted, risk-based, and subgroup-specific approaches should be prioritised in MASLD research, as the disease presents with a complex interaction of multiple cardiometabolic risk factors. A *Lancet Diabetes & Endocrinology* Series has highlighted the potential for precision-based approaches in cardiometabolic diseases focused on disease prediction and risk stratification approaches, which can be cost-effective by providing specific management strategies. Low-cost, easy-to-use risk stratification tools like the Indian Diabetes Risk Score could be valuable for prioritising screening and management. Considering the limited accuracy of imaging techniques like ultrasonography in isolation, it will be prudent to develop composite scores combining different parameters including inflammatory biomarkers; some studies show promising results in this regard.

At *The Lancet Regional Health – Southeast Asia*, we are keen to publish research that advances understanding of MASLD and other non-communicable diseases (NCDs). We particularly encourage evidence on epidemiology, natural history, screening and diagnostic approaches, risk prediction models, and management strategies which have the potential to inform specific policies to address the growing burden of MASLD and NCDs in the region.

